# Relationship between guilt and shame and depressive symptoms in normal population and patients with personality disorders

**DOI:** 10.1192/j.eurpsy.2021.1174

**Published:** 2021-08-13

**Authors:** O. Khokhlova, M. Vinogradova

**Affiliations:** 1 Department Of Neuro- And Pathopsychology, Faculty Of Psychology, Lomonosov Moscow State University, Moscow, Russian Federation; 2 Psychology Department, Middlesex University Dubai, Dubai, United Arab Emirates

**Keywords:** Guilt, depressive symptoms, personality disorders, Shame

## Abstract

**Introduction:**

Shame and guilt are often discussed in their association with depression. However, there is a need in deeper understanding of relationship between these emotions and depressive symptoms in personality disorders, where affective patterns do not reach the level of clinical depression.

**Objectives:**

To examine the differences in shame and guilt levels in normal subjects and patients with personality disorders and their association with depressive symptoms.

**Methods:**

In total, 28 patients (M=36.07, SD=11.87) diagnosed with personality disorders and 76 (M=29.67, SD=8.87) healthy individuals were recruited to take part in this study. Patients and healthy controls had equivalent educational level. Participants were given two standardized tests: Beck Depression Inventory and Test of Self-Conscious Affect (TOSCA) – 3.

**Results:**

There were significant differences in levels of guilt between patients with personality disorders (M=64.79, SD=6.78) and healthy individuals (M = 59.92, SD = 11.86), t (102) = 2.603, p = .011. Patients also demonstrated higher levels of shame (M=47.86, SD=9.70) than the participants without diagnoses (M = 43.38, SD = 14.96), however, these differences were not significant t (102) = 1.47, p > .05. It was found that depressive symptoms in normal population but not in patients significantly correlated with levels of guilt (r(76) = .124, p <.01) and shame (r(76)=.188, p<.01).

**Conclusions:**

It might be assumed that shame and guilt play different roles in emotional sphere of healthy individuals and patients with personality disorders, being associated with depressive symptoms in norm and unrelated to depressive symptoms in personality disorders.

